# Fibronectin Conformations after Electrodeposition onto 316L Stainless Steel Substrates Enhanced Early-Stage Osteoblasts’ Adhesion but Affected Their Behavior

**DOI:** 10.3390/jfb15010005

**Published:** 2023-12-21

**Authors:** Séverine Alfonsi, Pithursan Karunathasan, Ayann Mamodaly-Samdjee, Keerthana Balathandayutham, Sarah Lefevre, Anamar Miranda, Olivier Gallet, Damien Seyer, Mathilde Hindié

**Affiliations:** 1 Laboratoire de Physicochimie des Polymères et des Interfaces (LPPI Lab), CY Cergy Paris University, F-95000 Cergy, France; severine.alfonsi@cyu.fr (S.A.); k_pithursan@hotmail.fr (P.K.); ayann.samdjee@icloud.com (A.M.-S.); keerthanabala89@gmail.com (K.B.); sarah.lefevre25@gmail.com (S.L.); 2 Equipe de Recherche sur les Relations Matrice Extracellulaire-Cellule (ERRMECe Lab), CY Cergy Paris University, F-95000 Cergy, France; anamar.miranda-jimenez@cyu.fr (A.M.); olivier.gallet@cyu.fr (O.G.); damien.seyer@cyu.fr (D.S.)

**Keywords:** 316L stainless steel, polypyrrole, electrodeposition, bio-functionalization, fibronectin coatings, STRO-1^+^ A pre-osteoblasts

## Abstract

The implantation of metallic orthopedic prostheses is increasingly common due to an aging population and accidents. There is a real societal need to implement new metal implants that combine durability, good mechanical properties, excellent biocompatibility, as well as affordable costs. Since the functionalization of low-cost 316L stainless steel substrates through the successive electrodeposition of a polypyrrole film (PPy) and a calcium phosphate deposit doped with silicon was previously carried out by our labs, we have also developed a bio-functional coating by electrodepositing or oxidating of fibronectin (Fn) coating. Fn is an extracellular matrix glycoprotein involved in cell adhesion and differentiation. Impacts of either electrodeposition or oxidation on the structure and functionality of Fn were first studied. Thus, electrodeposition is the technique that permits the highest deposition of fibronectin, compared to adsorption or oxidation. Furthermore, electrodeposition seems to strongly modify Fn conformation by the formation of intermingled long fibers, resulting in changes to the accessibility of the molecular probes tested (antibodies directed against Fn whole molecule and Fn cell-binding domain). Then, the effects of either electrodeposited Fn or oxidized Fn were validated by the resulting pre-osteoblast behavior. Electrodeposition reduced pre-osteoblasts’ ability to remodel Fn coating on supports because of a partial modification of Fn conformation, which reduced accessibility to the cell-binding domain. Electrodeposited Fn also diminished α5 integrin secretion and clustering along the plasma membrane. However, the N-terminal extremity of Fn was not modified by electrodeposition as demonstrated by *Staphylococcus aureus* attachment after 3 h of culture on a specific domain localized in this region. Moreover, the number of pre-osteoblasts remains stable after 3 h culture on either adsorbed, oxidized, or electrodeposited Fn deposits. In contrast, mitochondrial activity and cell proliferation were significantly higher on adsorbed Fn compared with electrodeposited Fn after 48 h culture. Hence, electro-deposited Fn seems more favorable to pre-osteoblast early-stage behavior than during a longer culture of 24 h and 48 h. The electrodeposition of matrix proteins could be improved to maintain their bio-activity and to develop this promising, fast technique to bio-functionalize metallic implants.

## 1. Introduction

Implantable materials, such as metallic orthopedic prostheses, continue to be increasingly used due to both an aging population and traumatology. Orthopedic prostheses, 70% of which are made from metals [1], constitute a flourishing market of USD 115 billion, which is still growing [2]. Metallic materials are first used as bone implants due to their physical and mechanical properties and inert nature [3]. The three most implanted metallic biomaterials are titanium alloys (Ti), cobalt–chromium alloys, and medical-grade stainless steel (316L SS). 316L SS plates, screws, and pins are extensively used as bone fracture fixation devices due to their mechanical properties and low cost [4]. However, corrosion is the main reason for 316L SS implants’ failure when they come into contact with biological fluid [5]. The release of metal ions induced by corrosion causes aseptic loosening, osteolysis, adverse local tissue reactions, or inflammation of the area around metal implants [6]. Despite its high cost, biomedical-grade titanium (Ti) is widely used in biomedical implants such as joint replacements, bone plates, and dental implants [7] thanks to its biocompatibility and resistance to corrosion. However, the release of Ti oxide from Ti alloys induces allergy and exposure to toxic Ti nanoparticles which have been proven by in vitro studies [8].

Nevertheless, bacterial infections constitute another major cause of metallic prosthesis implantation failure. In periprosthetic infections, *Staphylococcus aureus* bacteria are frequently involved because of their high adaptation to human physiology and their ability to form biofilms that are tolerant to antibiotics [9].

Several physical and chemical techniques have been developed to protect 316L SS against corrosion such as trimethylsilane plasma nanocoatings [10], surface modification with deposition of a polycaprolactone film [11], nanosecond laser treatment of 316L SS surface [12], or sol–gel spin coating [13], but these techniques prevent the deposition of biodegradable coatings [14]. In this study, the approach used to prevent the 316L SS support from corroding is the electrodeposition of a conductive polymer film, which is a well-known approach [15]. Polypyrrole (PPy), polyaniline (PA), and poly (3,4-ethylene-dioxyethiophene) (PEDOT) are three of the conductive polymers most used for the protection of active metals such 316L SS from corrosion [16]. Furthermore, Martins et al. demonstrated that electrodeposition maintains the chemical composition and thickness of the coating, so coating depositions are reproducible [17]. Electrodepositions are reproducible even on supports with complex geometry or on porous surfaces. In addition, only inexpensive equipment is needed to perform these depositions [18].

The conducting polymer chosen for this study was PPy because of its high resistance to corrosion and delamination [19], easy synthesis, high conductivity, and good biocompatibility [20,21]. PPy-based materials were described with levels of immunogenicity that are comparable to other FDA-approved biomaterials [22]. Still, PPy coating improvements were necessary to increase their biocompatibility and adherent cells’ adhesion. Inorganic components were incorporated in PPy coatings, such as hydroxyapatite nanoparticles or zinc oxide particles, to improve biocompatibility with the bone matrix [23,24]. However, the absence of PPy functional groups such as carboxyl groups [25] able to interact with human body biomolecules blocks the further adsorption of exogenous biological molecules such as matrix proteins [26]. Several approaches have recently been developed to improve PPy biocompatibility such as RGD peptide covalent grafting via cysteine residues [27].

Cellular matrix (ECM) proteins, such as fibronectin (Fn) or collagen, are important for early-stage adhesion, migration, and differentiation, and they are used to improve implants’ bio-activity [28,29]. Thus, the bio-functionalization of implants with Fn was shown to stimulate the adhesion of cells such as osteoblasts [30]. ECM proteins immobilized on supports facilitate the indirect formation of bone tissue. These proteins create ECM adhesion sites, allowing osteoblastic cells to attach and initiate the osseointegration process [31].

Different methods were employed to bio-functionalize implants with Fn such as adsorption [32], grafting [33], or microcontact printing [34]. The choice of the most appropriate technique depends on the physico-chemical properties of the implant on which functionalization is performed [35]. Since the biological inertia of the PPy surface reduces cell affinity and bioactivity [36], an alternative method of protein bio-functionalization was employed to improve the bioactivity of our implants coated with a PPy film: Fn electrodeposition.

The aim of this present work was to optimally functionalize 316L SS supports through successive electrodepositions of a PPy film and Fn. The coatings’ physico-chemical properties were characterized and the effects of electrodeposition on Fn bioactivity were analyzed.

The response of the STRO-1^+^ A pre-osteoblasts cultured on our supports was also studied on early-stage cultures as well as longer ones in order to investigate the effect of electro-coating of Fn on cell bioactivity and morphology.

## 2. Material and Methods

### 2.1. Support Preparation

#### 2.1.1. Steel Plate Treatment

Medical-grade 316L SS plates (Arcelor, La Plaine Saint-Denis, France) were cut using blue-laser technologies (CMS, Bressuire, France) with a L3030 laser cutting machine (Trumpf, Villepinte, France) to obtain supports with a precise size of 30 × 5 × 1 mm.

Before each electrochemical experience, substrates were polished with abrasive paper using grain sizes 800 Si-C and a Struers LaboPol-1 polisher. The supports were then cleaned through immersion for 15 min at room temperature in an ultrasonic cleaner sequentially containing acetone, ethanol, and ultra-pure water. They were also air-dried.

#### 2.1.2. PPy Coating on 316L SS

The electrochemical polymerization of Py on the 316L SS plates was performed using a three-electrode potentiostat/galvanostat (VSP 150, Bio-logic Scientific Instrument, Seyssinet-Pariset, France). The 316L SS support was used as a working electrode, a saturated calomel electrode (SCE) as a reference, and a steel grid as a counter-electrode. The electrolytic solution used contained 0.2 M freshly distilled pyrrole (Thermo Fisher Scientific, Illkirch, France) and 0.5 M salicylate sodium (Sigma-Aldrich, Saint Quentin Fallavier, France).

The applied potential was scanned between −0.5 and 1.1V/SCE for 15 cycles at a fixed scan rate of 50 mV/s, and then rinsed with deionized water to remove unused monomer molecules prior to drying in the air. Each PPy layer formed on supports has a surface of 1.3 cm^2^.

### 2.2. Surface Characterization

#### 2.2.1. Profilometric Analysis

The roughness and thickness of surface deposits were determined using profilometry (DEKTAK 150, Palaiseau, France). The mean of three measures was calculated for over five different areas onto each support.

#### 2.2.2. Measure of the Surface Wettability

This measurement was performed using a Theta Flow goniometer (Biolin Scientific, Les Ulis, France). The goniometer helps measure the contact angle formed by a 5 µL drop of water deposited on the surface of a support. The surface angle contact was determined by measuring five different areas per support and it was performed on three different supports for each type.

### 2.3. Support Bio-Functionalization

#### 2.3.1. Fn Adsorption

Supports covered with a PPy film were immersed in an Eppendorf (Montesson, France) tube^®^, containing Fn diluted at 10 µg/mL in an adsorption buffer (HEPES 10 mM, NaCl 150 mM, and CaCl_2_ 15 mM, Sigma-Aldrich), for 10 min at 37 °C. Substrates were then rinsed with an adsorption buffer and stored at 4 °C.

#### 2.3.2. Fn Oxidation

Adsorbed Fn on supports was oxidized by applying an electric current through chronoamperometry. The electrochemical assembly was the same as the one used previously for PPy electrodeposition. Oxidation consisted of applying a potential of +0.25 V/EOC for 10 min with an adsorption buffer used as an electrolyte. The substrate was then rinsed twice with adsorption buffer and stored at 4 °C.

#### 2.3.3. Fn Electrodeposition on Support

Fn was directly electrodeposited onto the supports through chronoamperometry using the same 3-electrode assembly. Electrodes were immersed in 20 mL of adsorption buffer containing Fn at a concentration of 10 µg/mL. The same parameters used for oxidation were applied, at a potential of +0.25 V/EOC for 10 min. The support was then rinsed and stored at 4 °C as described previously.

### 2.4. Study of Fn Behavior on Each Type of Support

#### 2.4.1. Immunostaining of Fn-Deposited Coatings on Supports

The three types of samples were treated with a blocking buffer prepared with phosphate-buffered saline (PBS) containing 5% of bovine serum albumin (BSA) (Sigma-Aldrich) for 30 min at room temperature to block non-specific binding sites. The supports were then incubated for 1 h at room temperature with a 1/1000 dilution of anti-Fn rabbit polyclonal antibody (F3648, Sigma) in saturation buffer. After being rinsed with 0.1% (*w*/*v*) PBS–BSA buffer (3 times for 5 min), supports were incubated for 1 h with an Alexa Fluor 488-coupled anti-rabbit antibody (Thermo Fisher Scientific, Illkirch, France) at 1/200 in saturation buffer. After rinsing, observations and image capture were conducted using a Confocal Laser Scanning Microscope (CSLM) LSM 900 (Zeiss, Rueil Malmaison, France). Image post-processing was performed using the Fiji^®^ software (2.9.0 version).

#### 2.4.2. Quantification of Fn Present on Supports

A desorption of Fn present on supports was first performed to enable protein assays. Fn-coated supports were desorbed with 500 µL of adsorption buffer containing 1% SDS (*v*/*v*) 3 times for 5 min in an ultrasonic bath. An amount of 150 µL of the desorbed Fn solution from each sample was transferred into a 96-well plate with 150 µL of working solution from a QuantiPro^®^ BCA Assay kit (Sigma-Aldrich). The plate was then incubated for 1 h at 60 °C. After allowing the samples to cool down, the absorbance of each well was read at 562 nm using a Xenius spectrophotometer (SAFAS, Monaco, Monaco).

With the help of a calibration curve obtained by measuring the absorbance for a known protein concentration range for Fn (linear curve in the concentration range studied), it was possible to determine the amount of protein in the solution after desorption.

#### 2.4.3. Enzyme-Linked Immunosorbent Assay (ELISA)

The accessibility of the Fn cell-binding domain was tested using ELISA with specific antibodies. Samples with either adsorbed, oxidized, or electrodeposited Fn were blocked for 1 h at room temperature with Tris buffer saline (TBS) containing 1% BSA (*w*/*v*) and 0.1% Tween20 (*v*/*v*) (blocking buffer). Incubation with a monoclonal antibody against the Fn cell-binding domain (sc18825 mouse IgG Santa Cruz, Dallas, TX, USA) 1/1000 diluted in blocking buffer was then performed. After 60 min of incubation at room temperature, samples were washed 3 times with Tween^®^ 0.1%-TBS (T-TBS). Then, secondary antibody anti-mouse IgG-peroxidase (A4416 Sigma-Aldrich) 1/5000 diluted in blocking buffer was incubated for 1 h at room temperature. Three washes with T-TBS were performed, and the remaining antibodies were revealed with a tetramethylbenzidine substrate for five min. The reaction was stopped with 0.5 M sulfuric acid, and measurements were performed at 405 nm using a Xenius micro-plate reader (SAFAS).

### 2.5. Cellular Influence of Supports

The samples were first sterilized for 1 h at room temperature in sterile PBS with 100 units/mL of penicillin (Gibco, Thermo Fisher Scientific, Illkirch, France) and 0.1 mg/mL of streptomycin (Gibco). The supports were rinsed twice with sterile PBS and then dried for 30 min at 37 °C before cell culture.

#### 2.5.1. Human Cell Adhesion on Supports

F/STRO-1^+^ A (STRO) osteoblast progenitor cells were kindly provided by Dr. P. Marie (Inserm U1132, Paris Diderot University, Paris, France). They were cultured at 37 °C in Iscove’s modified Dulbecco’s medium (IMDM) (Gibco) containing 10% (*v*/*v*) fetal bovine serum (FBS), 2 mM Glutamax^®^ (Invitrogen, Thermo Fisher Scientific, Illkirch, France), 100 units/mL of penicillin (Gibco), and 0.1 mg/mL of streptomycin (Gibco). Cells were incubated at 37 °C in a humidified atmosphere (95% relative humidity) with 5% CO_2_ until pre-confluence.

#### 2.5.2. Bacteria Adhesion on Supports

The reference *Staphylococcus aureus* CIP4.83 strains were purchased from the “Collection de l’Institut Pasteur Paris”. Bacteria were grown in Tryptic Soy broth (TSB, BD™ Biosciences, San Jose, CA, USA) for 24 h at 37 °C.

The bacterial suspension was quantified with a spectrophotometer (ThermoSpectronic^®^ Genesys 20) at a wavelength of 600 nm. The bacterial suspension was diluted in PBS to obtain 10^5^ CFU/mL. Supports were put in a tube containing 500 µL of bacterial suspension and incubated at 37 °C in constant agitation for 3 h. The supports were then rinsed twice with 0.9% (*w*/*v*) NaCl solution (SW) and placed in an Eppendorf^®^ tube with 500 µL of SW to be treated with sonication for 10 min. The mix of bacteria and SW was preserved. This sonication treatment was renewed twice. The total supernatant obtained was diluted (serially diluted 1:10) and 100 µL of each suspension was deposited on a TS agar plate and incubated at 37 °C for 24 h. A count of the colonies obtained was performed to determine the quantity of adherent bacteria on each type of support.

#### 2.5.3. Mitochondrial Activity

Pre-confluent STRO cells were treated with trypsin/EDTA, harvested through centrifugation, and the cell pellet was re-suspended in IMDM containing 10% FCS and 1% Glutamax^®^. An amount of 50 µL of cell suspension was seeded on each substrate (corresponding to 20,000 cells/cm^2^). Culture was performed for 24 or 48 h in a humidified atmosphere of 5% CO_2_, at 37 °C. After incubation time, each support was transferred to an Eppendorf tube with 500 µL of culture medium and 50 µL of AlamarBlue^TM^ reactive (Thermo Fisher Scientific, Illkirch, France). Tubes were incubated for 3 h at 37 °C, and 100 µL of media from each tube was transferred to a 96-well black plate in order to read fluorescence with a Xenius spectrofluorometer (SAFAS) using a fluorescence excitation wavelength of 560 nm and an emission of 590 nm. The measurements of mitochondrial activity were renewed in three independent experiments in duplicate.

#### 2.5.4. Cell Proliferation

To determine cell proliferation, previous samples were fixed for 15 min with 4% paraformaldehyde (Sigma-Aldrich) solution in PBS. Cells were then labeled for 30 min with a solution containing 10 μg/mL of 4′,6-diamidino-2-phenylindole dihydrochloride (DAPI, Sigma-Aldrich). Cellularized supports were examined using a Leica fluorescence microscope (10× objective). For each sample, 3 independent fields were analyzed. Data are representative of three different experiments performed in duplicate. Image post-processing was performed using the Fiji^®^ software (2.9.0 version).

#### 2.5.5. Cell Morphology

Pre-osteoblasts were seeded on sterile supports as described previously, and they were grown for 3 h, 24 h, or 48 h. Cells were first fixed with a 4% paraformaldehyde solution for 10 min and rinsed with PBS. Cells were then permeabilized with a 0.2% (*v*/*v*) PBS-Triton solution for 5 min, and the supports were rinsed with PBS. Each support was placed for 30 min in a blocking buffer, PBS–BSA 5% (*v*/*m*). Supports were incubated for 1 h at room temperature with either a primary antibody anti-Fn rabbit (F3648, Sigma Sigma-Aldrich Saint Quentin Fallavier, France) diluted at 1/200th in blocking buffer, a primary antibody anti-α5 integrin (MAB 33231 Invitrogen) diluted at 1/100th, or a primary antibody anti-Fn extra domain A (EDA) (BML-FG6010, Enzo, Lyon, France) 1/100th diluted in blocking buffer. They were then rinsed with PBS–BSA 0.1% (*v*/*m*) buffer and incubated for 1 h with a secondary antibody (anti-IgG rabbit AlexaFluor 488 diluted at 1/400th, or anti-IgG mouse AlexaFluor 488 diluted at 1/400th) with DAPI (10 μg/mL) and AlexaFluor 568-coupled phalloidin (1/400th diluted) (Thermo Fisher Scientific, Illkirch, France). After rinsing, a lamella was fixed on each sample with Prolong Gold^®^. Observations and image capture were conducted using a CLSM, LSM 900 (Zeiss) with objectives (×20, or ×63). For each sample, 3 independent fields were analyzed. Post-treatment images were taken using the Fiji^®^ software.

### 2.6. Statistical Analysis

Data were analyzed using the Instat3^®^ statistical software (GraphPad Software, 3 version). The statistical significance between groups was assessed, after the normality test was passed, using one-way analysis of variance (ANOVA), followed by Student–Newman–Keuls multiple comparison tests for protein assays, mitochondrial activity tests, and cell proliferation or Dunn test for ELISA. Experimental results are expressed as means ± standard deviation at a significance level of *p* < 0.001, *p* < 0.05, or *p* < 0.01.

## 3. Results and Discussion

### 3.1. Bio-Functionalization and Characterization of Supports

#### 3.1.1. Electrodeposition of PPy Coatings

**Figure A1:**
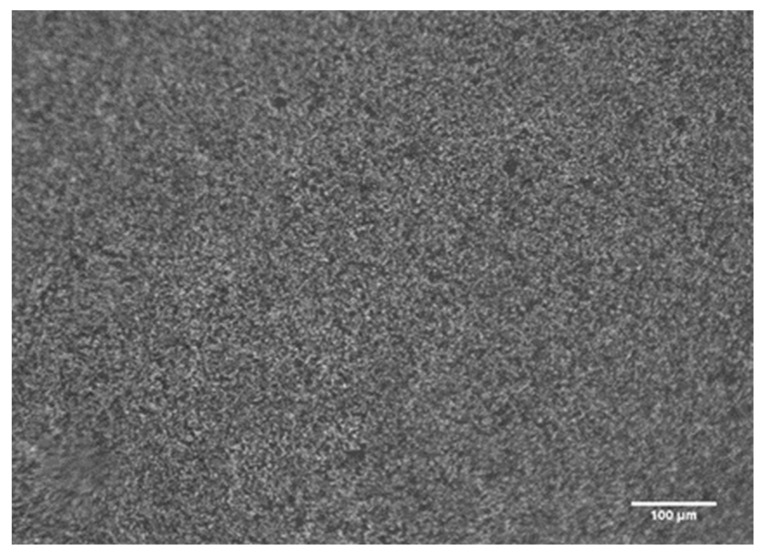
Observation of PPy coating on support with an optical microscope.

After applying the potential between −0.5 and +1.1V/ECS with a scanning speed of 50 mV/s for 15 cycles, a black smooth and adherent film of PPy was obtained on 316L SS supports. The uniformity of the PPy film was controlled through optical microscopy observations (Appendix A, Figure A1).

In order to analyze the PPy films formed on the supports, a profilometry study was performed to determine their thickness and roughness. PPy films had a thickness of 4.98 µm ± 0.15 µm and a low roughness of 0.27 µm ± 0.05 µm. In this study, the films obtained were thicker (4.9 µm vs. 2.2 µm) than those obtained by Hamdaoui S et al. [37]. These results are consistent because the Py solution used was twice as concentrated as the one previously used in the lab.

#### 3.1.2. Surface Wettability of Supports

Support water contact angle was measured before and after PPy film deposition. The 316L SS substrates presented a wettability of 100 ± 3°, so they were considered hydrophobic, whereas the substrates covered by PPy had a more hydrophilic wettability of 79 ± 4°. Hydrophobic surfaces induce a conformational change in Fn by reducing accessibility to the cell-binding domain (CBD) [29,38] and preventing cell adhesion [39]. Hence, wettability values measured for PPy supports were more favorable to the adsorption of matrix proteins such as Fn or vitronectin and improved the subsequent adhesion of cells cultivated on their surface.

#### 3.1.3. Support Functionalization with Fn

Three different methods were employed to deposit Fn on supports covered by a PPy film:

- AD: simple adsorption of Fn.
- OX: oxidation of precoated Fn.
- ED: electrodeposition of Fn.

A potential of +0.25 V/EOC for 10 min was applied on supports coated by Fn to prepare Ox supports. Chronoamperometry with the same potential used for oxidation was employed to induce the electrodeposition of Fn on PPy films. This functionalization of Fn through electro-oxidation or electro-deposition was compared to a simple adsorption of Fn. The presence and morphology of Fn on supports were determined using confocal laser scanning microscopy (CSLM).

### 3.2. Study of the Bioactivity of Fn Deposited on Each Type of Supports

#### 3.2.1. Quantity of Fn on Substrates

**Figure 1:**
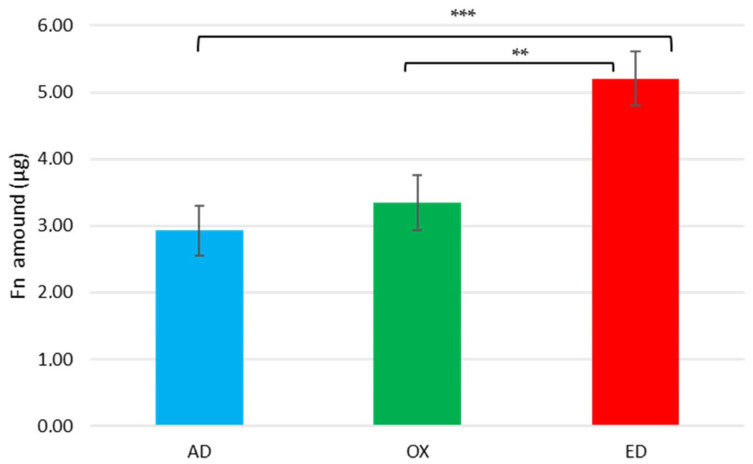
Surface protein quantities after 1% SDS rinses for the Fn initially adsorbed (AD), oxidized (OX), or electrodeposited (ED) on PPy-coated 316L SS supports. Values are means ± standard error means. Data are representative of three independent experiments performed in triplicate; significant differences were determined using a one-way ANOVA analysis, and the *p*-value of the test is 0.0004 (** *p* < 0.01, *** *p* < 0.001).

**Figure A2:**
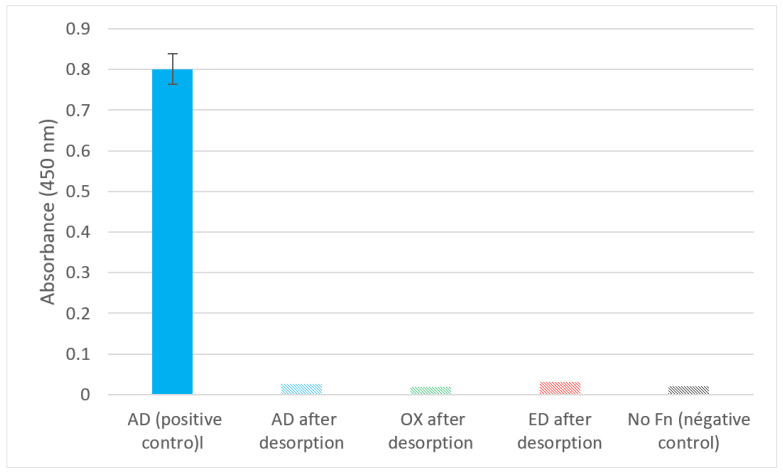
ELISA test of Fn adsorbed (AD), oxidized (OX), or electrodeposited (ED) on PPy-coated 316L SS substrates after desorption using a polyclonal antibody targeting Fn. Positive control was performed with Fn adsorbed (AD) without desorption, and negative control was performed with substrate without Fn coating.

The quantity of Fn present on the three types of supports was determined using a BCA assay after protein desorption with 1%SDS solution. Controls were performed to confirm the total desorption of proteins by this treatment (Appendix A, Figure A2). In Figure 1, the results of protein quantification show a mean amount of 2.9 µg ± 1.7 µg proteins adsorbed on AD supports, 3.3 µg ± 2.1 µg proteins on OX supports, and 5.2 µg ± 2.1 µg proteins on ED supports. Compared to AD supports, a significant increase of 78% was measured on ED supports after protein assays. No significant differences were detected between AD and OX supports. The quantity of deposited Fn through electrodeposition seems to be strongly higher than the one after simple adsorption and it was confirmed by statistical analysis. Different results were observed with pronectin, a synthetic protein electrodeposited on titanium supports [40], but the electrodeposition of macromolecules depends on their isoelectric points [41]. The Fn (with an isoelectric point at 5.5) was negatively charged in an adsorption buffer at pH 7.5 so it may interact more easily with PPy films that are positively charged [42]. Our hypothesis is that the deposition of Fn was enhanced on ED supports by their surface charge and the potential applied for electrochemical deposition.

**Figure 2:**
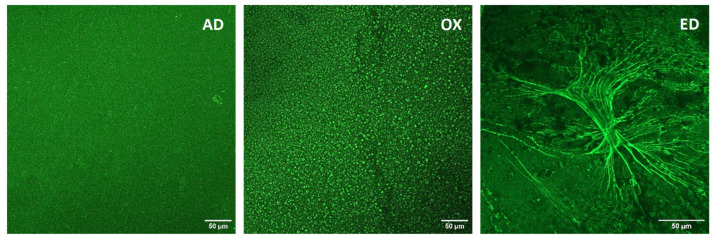
Images of Fn adsorbed (AD), oxidized (OX), or electrodeposited (ED) on PPy-coated 316L SS supports. Fn was immunostained to be observed by CSLM. Scale bar: 50 μm. Data are representative of two different experiments.

The Fn was stained with an anti-Fn polyclonal antibody and an Alexa Fluor^®^ 488-coupled secondary antibody (green). CSLM images of Fn are shown in Figure 2: on AD substrates, a homogenous coating of Fn is observed, whereas on OX supports, Fn microspheres are observed. A totally different organization is visualized on ED supports, which present long Fn fibers organized along fractal structures (Figure 2). The morphology of Fn observed on AD supports is similar to the ones described after Fn coating on supports such as glass coverslips [43] or titanium substrate [44]. Fn aggregates observed on OX substrates are comparable to the ones described by Bascetin R. et al. [45] after high-temperature treatment of Fn; this indicates denaturation of Fn and aggregation patterns during the oxidation step on OX supports.

#### 3.2.2. Biological Conformation of Fn

**Figure 3:**
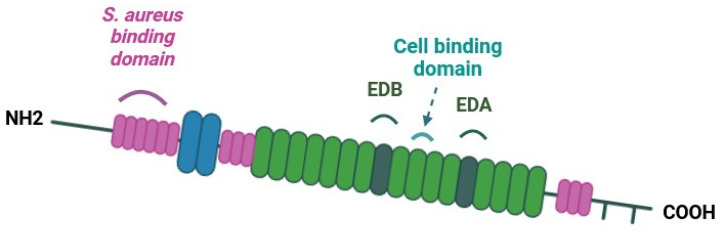
Schematic representation of fibronectin monomer. Binding sites for *Staphylococcus aureus* bacteria and the integrin α5β1/cell-binding domain are reported. The extra domain A (EDA), which is involved in matrix remodeling, and the extra domain B (EDB), which has an important role during embryogenesis, are also indicated.

**Figure 4:**
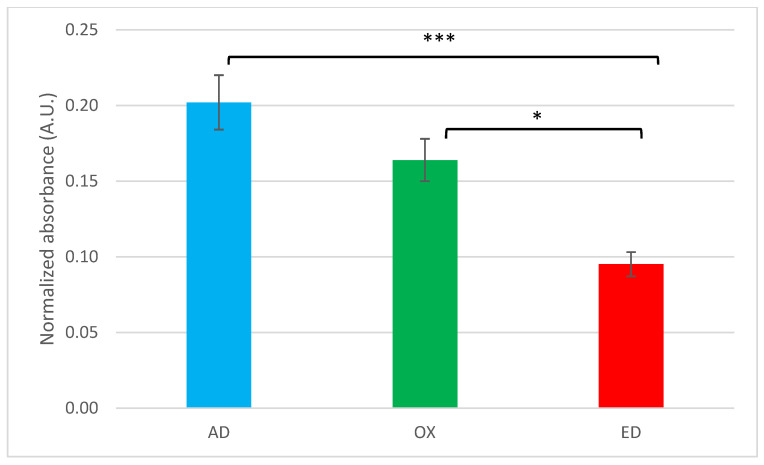
ELISA test of Fn adsorbed (AD), oxidized (OX), or electrodeposited (ED) on PPy-coated 316L SS substrates using monoclonal antibodies specific to the assessment of cell-binding domain accessibility. Values are means ± standard error means (*n* = 3, * *p* < 0.05, *** *p* < 0.001).

The bioactivity of Fn on the supports is indirectly determined by Fn adhesion capacity due to the preservation or not of the cell-binding domain in the structural conformation of Fn. Fn is a ≈450-kDa dimeric glycoprotein with a modular structure [46]. In Figure 3, a schematic representation of a Fn monomer shows the organization of its specific domain such as the CBD. Thanks to ELISA tests, targeting Fn CBD, only non-denaturated Fn could be detected. Antibodies used are directed against the conformational cell-binding domain of Fn. The absorbance measured is proportional to the quantity of cell-binding domain available. The strongest signal was detected on AD supports (Figure 4), meaning that the CBD of Fn (domains III9 and III10) is more accessible when Fn is absorbed. Compared to AD supports, accessibility to the CBD of Fn is lower on OX and ED supports. If data measured are normalized with the quantity of Fn measured on supports and compared to AD supports, it appears that Fn CBD is, respectively, 19% and 42% less accessible on OX supports and ED supports. These decreases can be explained by a conformational change in Fn resulting from oxidation or EPD. Similar results were described with the formation of Fn aggregates after thermal and mechanical denaturation. This treatment induced a modification of conformational organization that decreased Fn CBD accessibility [45].

### 3.3. Effects of Supports on Pre-Osteoblast Early-Stage Behavior

#### 3.3.1. Cell Morphology

**Figure 5:**
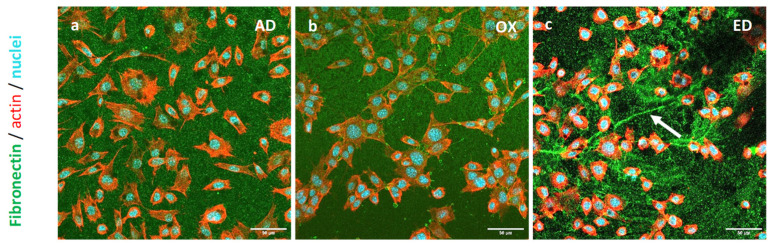
Images of STRO-1^+^ A cells cultured on Fn adsorbed in (**a**) AD, oxidized in (**b**) OX, or electrodeposited in (**c**) ED on supports for 3 h. Fn, nucleus, and actin cytoskeleton are stained in green, blue, and red respectively. Scale bar: 50 μm.

The morphology of pre-osteoblasts was observed via confocal laser scanning microscopy (CLSM). Cells were cultured for 3 h on the three types of supports before being fixed. Fn filaments were stained with a polyclonal antibody directed against Fn (green), nuclei with DAPI (blue) as the DNA probe, and the actin cytoskeleton with Alexa Fluor^®^ 568 phalloidin (red). Pre-osteoblast CSLM images are shown in Figure 5: on AD supports, cells were evenly spread out and had a clearly distinguishable actin cytoskeleton (Figure 5a). Cells were attached to all types of supports, but the typical aspect of the osteoblasts’ morphology was observed mostly on AD supports. A comparable number of cells was observed for all the supports. Pre-osteoblasts present a more elongated morphology on OX supports (Figure 5b) and are less spread out, while they are more rounded on ED supports (Figure 5c). A previous study showed similar results, where cells cultured on Fn aggregates were less spread out than the ones cultured on native Fn [47]. Pre-osteoblasts cultured on ED did not follow the Fn fiber and were the least spread out, which could be due to the reduced accessibility of Fn CBD. This phenomenon was also described with HUVEC cells cultured on self-assembled monolayers which decreased Fn CBD access [29].

#### 3.3.2. 5 Integrins and Cellular Fn Organization

**Figure 6:**
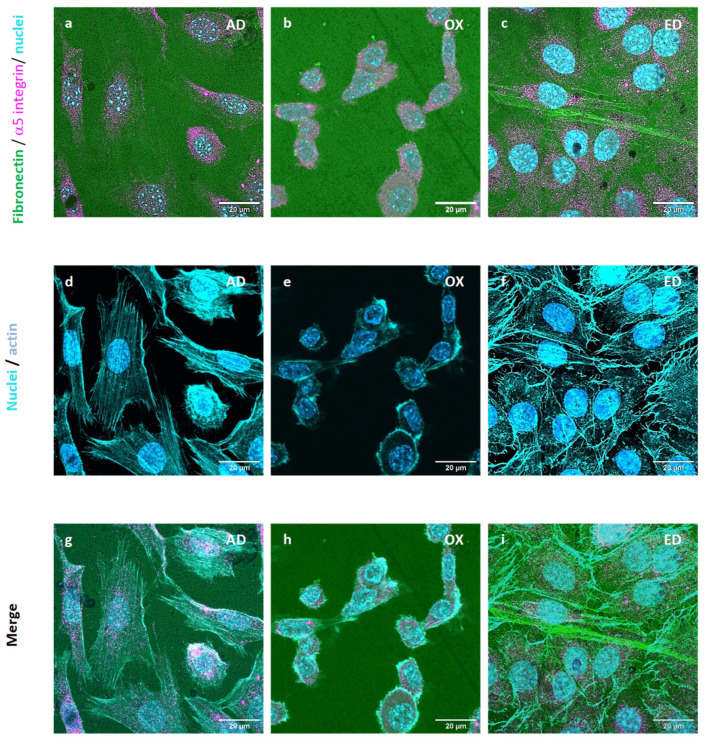
Cytoskeletal structure and cellular α5 integrin expression of STRO-1^+^ A pre-osteoblasts cultured for 3 h on supports functionalized by Fn adsorbed (AD) in (**a**,**d**,**g**) images, oxidized (OX) in images (**b**,**e**,**h**) or electrodeposited (ED) in images (**c**,**f**,**i**). Fn, actin cytoskeleton, α5 integrin, and nuclei are stained in green, cyan, magenta, and blue respectively. Scale bar: 20 μm.

The expression and organization of α5 integrins in pre-osteoblasts cultured for 3 h on supports were analyzed after immunostaining and observations using CSLM. Fn was stained with a polyclonal antibody against Fn (green) and α5 integrins monoclonal antibody targeting α5 integrins (magenta). Nuclei were labeled with DAPI (blue) and the actin cytoskeleton with Alexa Fluor^®^ 568 phalloidin (Thermo Fisher Scientific, Illkirch, France) (cyan). Pre-osteoblasts were cultured on the three types of supports; α5 integrins were observed in pre-osteoblasts each time but their organization was not the same for all supports. On AD supports, α5 integrins were around the nucleus and along the cell membrane (Figure 6g). Focal adhesion began to be organized on AD supports as we observed in previous work [48]. On OX supports, α5 integrins remained in the cytoplasm along the nucleus. Since α5 integrins specifically bind to Fn [49] at the CBD site (Figure 3), they probably are not able to bind to oxidized Fn with a CBD site that is less accessible. On ED supports, α5 integrins showed around the nucleus and a little in the cytoplasm (Figure 6c–i). A long Fn fiber was also observed on ED supports (Figure 6c), but no α5 integrin staining was detected along this structure. These results are consistent with ELISA tests results, demonstrating that the Fn CBD is less accessible on Fn fiber produced by electrodeposition treatment which induces less cell attachment.

**Figure 7:**
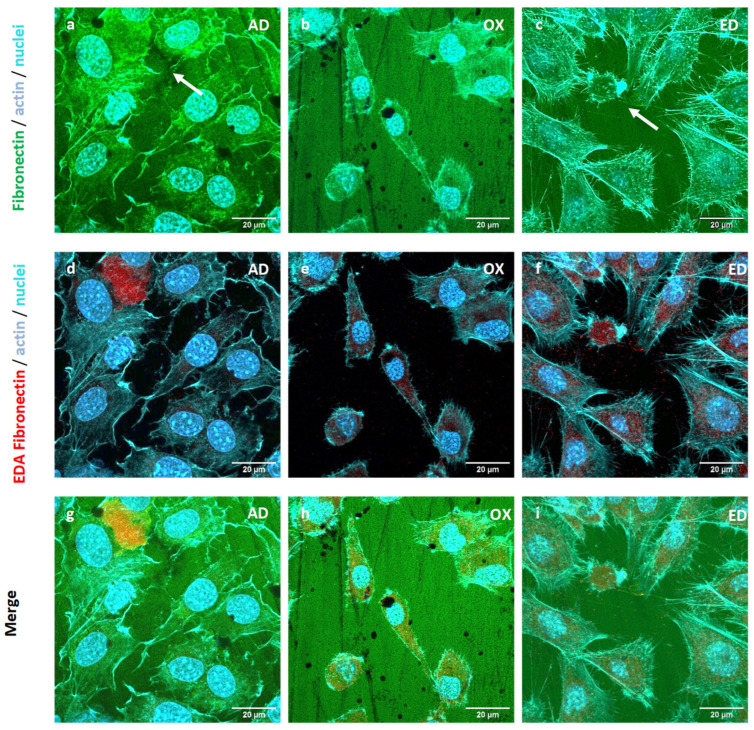
Cytoskeletal structure and Fn extra domain A (EDA) synthesis of STRO-1^+^ A pre-osteoblasts cultured for 3h on supports functionalized by Fn adsorbed (AD) shown in (**a**,**d**,**g**) images, oxidized (OX) in images (**b**,**e**,**h**), or electrodeposited (ED) presented in images (**c**,**f**,**i**). Fn, actin cytoskeleton, EDA Fn, and nuclei are stained in green, cyan, red, and blue respectively. Scale bar: 20 μm.

The expression of Fn extra domain A (EDA), which is specific to cellular Fn, was followed by immunological staining in pre-osteoblasts cultured on supports for 3 h. After culture and fixation cells were stained, fibronectin, EDA Fn, nuclei, and the actin cytoskeleton were stained with a polyclonal antibody against Fn (green), a monoclonal antibody targeting the EDA fragment of Fn (red), DAPI (blue), and Alexa Fluor^®^ 568 phalloidin (cyan), respectively. EDA Fn was observed in pre-osteoblasts cultured on the three types of supports. However, EDA Fn was secreted only by cell cultures on AD supports (Figure 7a). EDA Fn was detected only in the cytoplasm for cells cultivated on OX and ED supports. We previously described a synthesis of EDA Fn by STRO-1^+^ A cultured on plasmatic Fn coating and a cell remodeling of this coating [48]. EDA Fn is an insoluble Fn, known to participate in cell–matrix remodeling [50]. Only pre-osteoblasts cultured on AD supports have the ability to reorganize the Fn coating using Fn coating and EDA Fn jointly. Therefore, oxidation and electrodeposition modified the resulting Fn coating but also reduced EDA Fn secretion. We hypothesize that the important diminution in the Fn CBD and the reduction in EDA Fn secretion consequently inhibited Fn coating remodeling by pre-osteoblasts.

### 3.4. Early-Stage Bacterial Behavior on Supports

**Figure 8:**
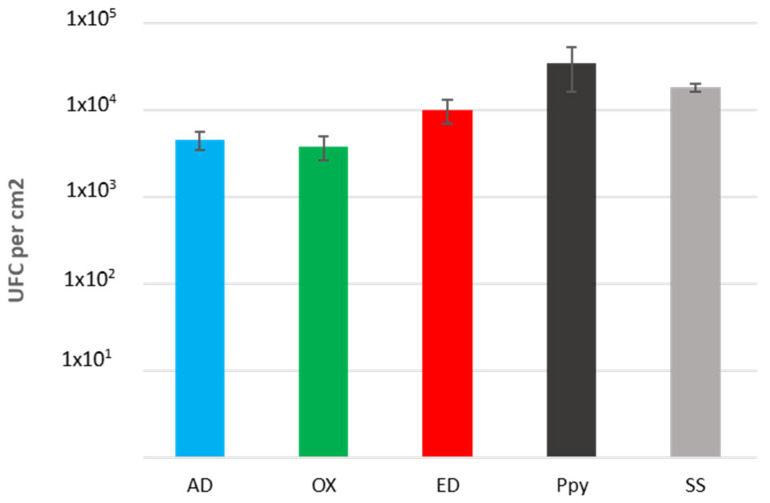
Bacterial attachment on bio-functionalized supports. Number of *Staphylococcus aureus* CIP4.83 bacteria adhering on Fn adsorbed (AD), oxidized (OX), electrodeposited (ED), polypyrrole (PPy) coating, or stainless steel (SS) substrate. The incubation time was 3 h. Values are means ± standard error means (*n* = 2).

All types of supports were deep in a *Staphylococcus aureus* bacteria suspension and incubated for 3 h at 37 °C. Adherent bacteria were detached using sonication, and bacteria obtained were diluted, seeded on an agar plate, and grown for 24 h at 37 °C to count the number of colonies formed on each support. No significant differences between each support were observed as shown in Figure 8. Comparable quantities of UFC were counted on AD, OX, and ED supports; however, more *S. aureus* seems to have adhered to supports coated only with a PPy film. Hence, Fn deposition methods did not appear to affect *S. aureus* adhesion on supports. These results seem to demonstrate that the *S. aureus* binding domain of Fn was not modified by either oxidation or electrodeposition.

Furthermore, at physiological pH, Fn is negatively charged [28]. The PBS buffer used for bacteria culture has a physiological pH, which maintains the Fn negative charge. On the other site, *S. aureus* presents a global negative surface charge [51]. Therefore, negatively charged *S. aureus* bacteria could interact more with the positive charge in the PPy structure than with the negative surface charge of Fn [52].

### 3.5. Pre-Osteoblast Behavior after Culture for 48 h on Supports

For longer pre-osteoblast cultures, we focused on AD and ED supports, which induced more specific cell responses as compared to OX supports, which induced a lack of cell attachment.

#### 3.5.1. Cell Morphology

**Figure 9:**
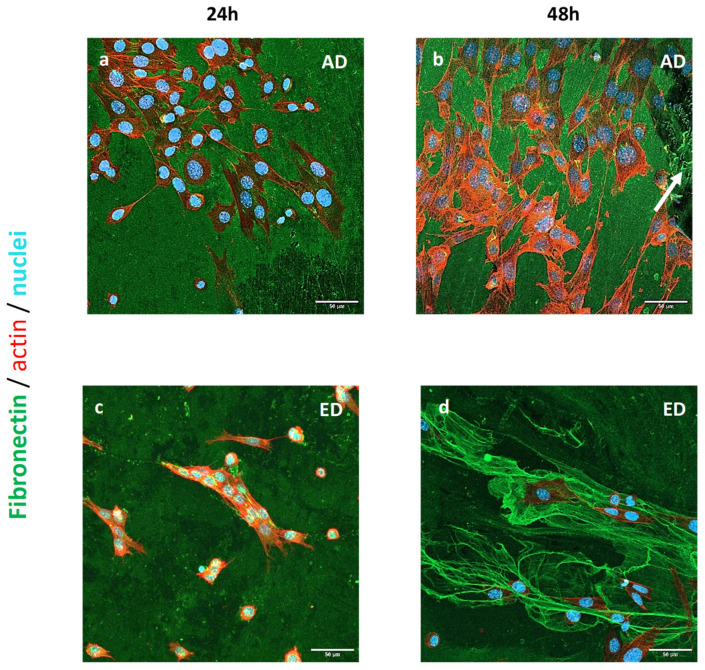
Images of STRO-1^+^ A cells cultured on Fn adsorbed (AD) or electrodeposited (ED) on supports for 24 h and 48 h. Fn, nucleus, and actin cytoskeleton are stained in green, blue, and red respectively. Scale bar: 50 μm.

The morphology of pre-osteoblasts cultured for 24 h and 48 h on both types of supports was studied after cell fixation and immunological staining as previously described. Fibronectin was labeled in green; nuclei and actin filaments were stained in blue and red, respectively. Pre-osteoblast CSLM images acquired are shown in Figure 9: on AD supports, cells had a clearly distinguishable actin cytoskeleton but they were less spread out than after 3 h culture and they had an elongated morphology (Figure 9a,b). On AD supports, pre-osteoblasts remodeled the Fn coating to produce fibril (arrow on Figure 9b). Cell cultivated on ED supports were also elongated but with a smaller area than on AD supports (Figure 9c,d). Pre-osteoblasts were grouped as clusters on ED (Figure 9c) or appeared entrapped in large Fn electrodeposited fibers (Figure 9d). On both ED supports, cells were less numerous than on AD supports. An elongated morphology and aligned actin fibers of pre-osteoblasts cultured on AD supports for 24 h are typical of those observed for osteoblasts cultured on Fn coating [53]. The lower quantity of cells observed on ED supports may be due to their inability to remodel the Fn matrix.

#### 3.5.2. Mitochondrial Activity

**Figure 10:**
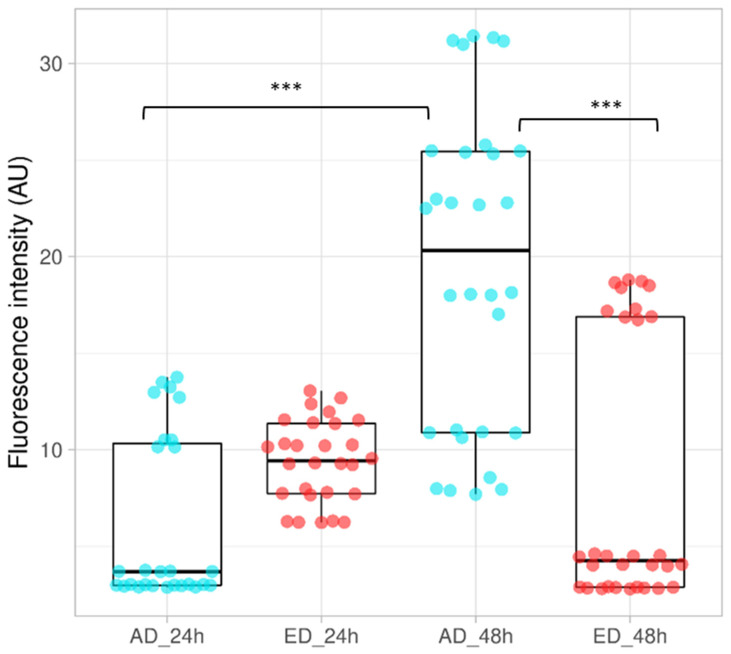
Mitochondrial activity of STRO-1^+^ A pre-osteoblasts cultured on Fn adsorbed (AD), or electrodeposited (ED) on supports for 24 h and 48 h. Activity measured for cells cultured on AD are represented by blue dots. Red dots are results obtained for cells cultured on ED substrates The box represents the data, with the horizontal line indicating the median value. (ANOVA test was performed, *n =* 3, *** *p* < 0.001).

Cells were cultured for 24 h and 48 h on both support types and an Alamarblue^TM^ (Thermo Fisher Scientific, Illkirch, France) assay was performed to determine their mitochondrial activity; results obtained are presented in Figure 10. After 24 h of culture, mean mitochondrial activity was higher on ED supports, but a significant augmentation of mitochondrial activity was observed on AD supports at t = 48 h. On the other hand, mitochondrial activity seemed to decrease on ED supports after 48 h of culture. These results may be due to a lower quantity of cells remaining on ED supports as observed in Figure 9d. Muhonen V et al. observed similar results with Fn coating on Nitinol supports [54]. The martensite Nitinol structure reduced the remodeling of Fn and induced a lower mitochondrial activity of murine pre-osteoblasts MC3T3-E1 after 48 h of culture compared to control.

#### 3.5.3. Proliferation of Pre-Osteoblasts

**Figure 11:**
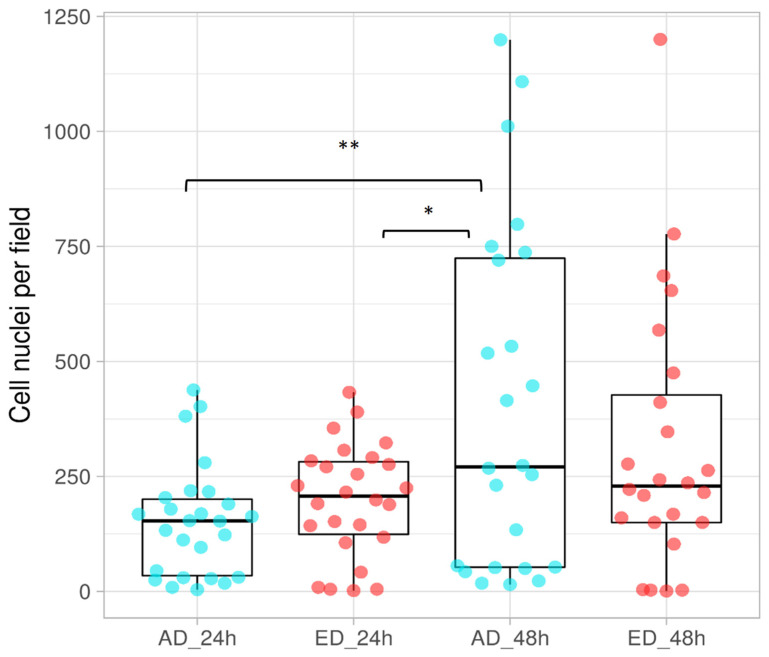
Proliferation of STRO-1^+^ A pre-osteoblasts cultured on Fn, adsorbed (AD), or electrodeposited (ED) on supports for 24 h and 48 h. Blue dots represent nuclei counted on AD supports and the red ones are the nuclei counted on ED ones. For each plot, the line within the box represents the median. (ANOVA test was done, *n =* 3, * *p* < 0.05, ** *p* < 0.01.).

Cells seeded on AD and ED substrates up to 48 h were fixed and their nuclei were stained with DAPI. Photos of three independent fields were taken and the number of nuclei was counted. The results obtained are presented in Figure 11. A significant augmentation of the nuclei number was observed between 24 h and 48 h on AD supports, whereas this number remained stable for ED supports. Therefore, pre-osteoblasts proliferated on AD substrates, whereas they did not on ED supports. These results are consistent with osteoblast morphology observations. Fibers formed through electrodeposition are not natural fibers and present less CBD accessibility than previously presented. This is why, as denatured fibers, they appear unable to stimulate cell attachment and further proliferation [55]. Furthermore, Fn remodeling is important for matrix organization and cell proliferation [56], which is not possible when Fn is electrodeposited. These difficulties for cells to adhere could induce a specific type of apoptosis in pre-osteoblasts named anoïkis. This phenomenon could explain the reduction in mitochondrial activity on ED supports after 48 h of culture [57].

## 4. Conclusions

Fn was successfully electrodeposited on supports and presented typical reproducible fractal fibrillar structures, whereas oxidized Fn formed aggregates. The amount of Fn electrodeposited on 316L SS supports was higher than the deposit performed through adsorption with or without electro-oxidation.

The early-stage culture of STRO-1^+^ A pre-osteoblasts on supports revealed adhesion and spreading on all types of support, suggesting the absence of toxicity. The culture of STRO-1^+^ A human pre-osteoblasts for 24 h and 48 h demonstrated good proliferation and mitochondrial activity on AD supports, whereas they were strongly inhibited on ED supports, probably due to the difficulty in remodeling the Fn matrix as observed previously [54]. It appeared that the Fn structure was structurally modified around its cell binding site, whereas the N-ter site remained functional.

In this study, an easy, fast, and reproducible way to bio-functionalize 316L SS supports was developed using electrodeposition. This approach to bio-functionalize metallic substrates is less time consuming than Fn grafting and more reproducible than Fn adsorption. This promising technique could also enable the simultaneous deposition of several biomolecules, such as an ECM protein and an anti-bacterial peptide, to improve implants’ properties.

## Data Availability

All the data used and analyzed for the current study are contained in the article.
